# Juvenile-specific high heat production contributes to the initial step of endothermic development in Pacific bluefin tuna

**DOI:** 10.3389/fphys.2025.1512043

**Published:** 2025-05-29

**Authors:** Takaaki K. Abe, Maho Fuke, Ko Fujioka, Takuji Noda, Hiroyuki Irino, Yoshikazu Kitadani, Hiromu Fukuda, Morten Bo Søndergaard Svendsen, John Fleng Steffensen, Takashi Kitagawa

**Affiliations:** ^1^ Atmosphere and Ocean Research Institute, The University of Tokyo, Chiba, Japan; ^2^ College of Bioresource Science, Nihon University, Fujisawa, Kanagawa, Japan; ^3^ Fisheries Resources Institute, Japan Fisheries Research and Education Agency, Yokohama, Kanagawa, Japan; ^4^ Department of Fisheries, School of Marine Science and Technology, Tokai University, Shizuoka, Japan; ^5^ Field Science Education and Research Center, Kyoto University, Kyoto, Japan; ^6^ Osaka Aquarium Kaiyukan, Osaka, Japan; ^7^ Marine Biological Section, University of Copenhagen, Helsingør, Denmark; ^8^ Graduate School of Frontier Sciences, The University of Tokyo, Chiba, Japan

**Keywords:** biologging, heat-budget model, metabolic rate, respirometry, red muscle development

## Abstract

Pacific bluefin tuna (*Thynnus orientalis*; PBT) can maintain their body temperature above ambient water (i.e., thermal excess) through high heat production and heat retention. The endothermic ability develops at 20–40 cm fork length ( $L_{f}$ ), which has been attributed to improved heat retention. Meanwhile, the contribution of heat-production capacity to the development of thermal excess is insufficiently understood. This study aimed to elucidate the ontogenetic pattern of heat production and its contribution to endothermic capacity in juvenile PBT using a heat-budget model (HBM) and swim-tunnel respirometry. The HBM was applied to 2–4 months of biologging data from juveniles (23–50 cm $L_{f}$ ; 200–4 kg) to estimate heat production rates ( ${\dot{T}}_{m}$ ), revealing that these rates remained high up to approximately 700 g and declined thereafter. Moreover, the comparison of the development of endothermic capacity with the HBM-estimated parameters suggests that in the early juvenile stages, when PBT rapidly develop the thermal excess, the high ${\dot{T}}_{m}$ contributes to the thermal excess. The high ${\dot{T}}_{m}$ in this stage implied the juvenile-specific development of aerobic capacity; therefore, metabolic rate and aerobic capacity-related tissues (red muscle and ventricle) were measured, and the scaling exponents were calculated in this stage (16–28 cm $L_{f}$ ; 50–420 g). Swim-tunnel respirometry was conducted on juvenile PBT in Japan (August–September 2022 and 2023), and the collected samples were used to measure red muscle and ventricular masses. The scaling exponents of tunas throughout life history are generally in the range of 0.6–0.9, while those for these traits were 1.0 or greater in this size range, supporting the juvenile-specific aerobic capacity development. In conclusion, this study reveals the ontogenetic characteristics of heat production-related traits in PBT and provides new insights into the developmental process of endothermic ability, beyond heat retention capacity.

## 1 Introduction

Animal body temperature is determined by internal heat production and heat exchange with the external environment (Schmidt-Nielsen, 1997; Butler et al., 2021). Each species possesses an optimal body temperature range and employs various strategies to maintain body temperature within this range (Butler et al., 2021). Based on their thermoregulation strategies, animals are classified as either endotherm or ectotherm. Endotherms sustain body temperatures above the surrounding environment through elevated metabolic heat production (Schmidt-Nielsen, 1997; Butler et al., 2021). In contrast, ectotherms do not retain their body temperature with their heat production; instead, they primarily rely on external heat sources, exploiting environmental thermal gradients to regulate body temperatures (Angilletta, 2009; Butler et al., 2021). Most fish are ectotherms: this is because the aquatic habitat is a challenging environment to maintain body temperature due to the high heat capacity of water, and the metabolic heat they produce is further lost through the gills and the skin. Nevertheless, among fish, a few species can maintain their body temperatures above ambient water, known as endothermic fish (Bernal et al., 2012; Wegner et al., 2015; Bernal et al., 2017). The endothermic ability is restricted to specific tissues/organs; therefore, it is referred to as “regional endothermy” to distinguish it from the “endothermy” observed in mammals and birds (Carey and Teal, 1969; Carey et al., 1971).

Tunas (tribe *Thunnini*) are notable examples of endothermic fish and have long been explored for their ability to maintain body temperatures (Kishinouye, 1923). Tuna species achieve their endothermic ability through both high heat production and retention capacity, and exhibit unique morphological traits associated with them. They possess a unique vascular arrangement around specific tissues/organs (e.g., red muscle, liver), where arteries and veins alternate (Kishinouye, 1923; Carey et al., 1971; Dickson and Graham, 2004). The vascular pattern, referred to as *rete mirabile*, functions as the counter-current heat exchangers to retain metabolic heat, and heat from venous blood returning to the heart is passed to arterial blood, thereby reducing heat loss at the gills.

Tuna species also exhibit high metabolic rates, generally measured by oxygen consumption rate ( ${\dot{M}}_{O_{2}}$ ), compared with ectothermic fish, reflecting their high metabolic heat production (Blank et al., 2007a). Moreover, they exhibit developed features conducive to aerobic metabolism, such as greater proportions of the ventricle and red muscle (Dickson et al., 2000; Graham and Dickson, 2001; Kubo et al., 2008). The red muscle serves as a major source of metabolic heat, and the ventricle allows the high oxygen demand of tunas. The red muscle arrangement along the body’s medial axis further reduces heat dissipation. The physio-morphological features of tuna are shared in endothermic sharks (lamnid sharks), demonstrating that these traits represent one valid solution to acquiring the endothermic ability for fish. However, the endothermic ability of tuna is not innate, which is statued throughout their ontogeny (Dickson, 1994; Dickson et al., 2000; Kubo et al., 2008; Malik et al., 2020; Kitagawa et al., 2022). Typically, the endothermic capability of tuna starts to represent the ability to maintain their body temperature from early juvenile (>20 cm fork length, $L_{f}$ ) (Dickson, 1994; Dickson et al., 2000). The developmental process of the endothermic ability has been well described in Pacific bluefin tuna (*Thynnus orientalis*; PBT).

**FIGURE 1:**
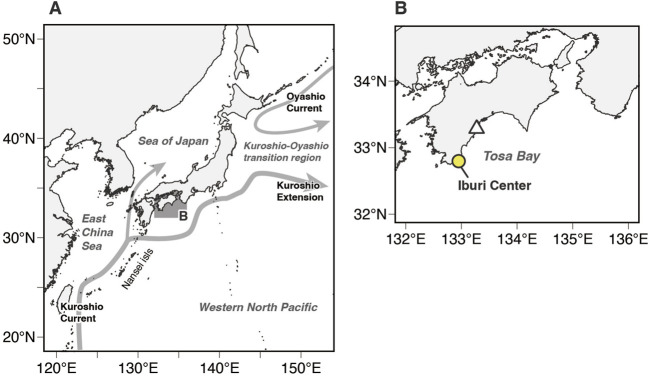
**(A)** Map of the western North Pacific Ocean, showing the study area (shaded area). Schematic of near-surface currents around Japan: Kuroshio Current, Kuroshio Extension, and Oyashio Current (gray arrows) **(B)** Enlarged map of the study area. The white triangle represents the release location of tagged Pacific bluefin tuna juveniles. The yellow-filled circle indicate the location of the Iburi Center, Osaka Kaiyukan Marine Biological Research Institute.

Pacific bluefin tuna and other bluefin tuna species, including Atlantic bluefin tuna (*Thunnus thynnus*) and southern bluefin tuna (*Thynnus maccoyii*), have well developed *retia mirabilia* among tuna species, and the adults generally show high heat retention capacity exceeding 10°C of thermal excess ( $T_{X}$ ) between body and water (ambient) temperatures ( $T_{b}-T_{a}$ ) when in relatively cold water. They can elevate the temperature of their locomotor muscle, viscera, brain, and eye tissues above that of water (Carey and Teal, 1966; 1969; Carey et al., 1971; Graham, 1995; Altringham and Block, 1997). With the prominent endothermic ability, these fish species have expanded their niches to low-temperature waters and improved their ability to sustain high-speed swimming due to their high aerobic metabolism and warm muscles (Stevens and Carey, 1981; Block and Finnerty, 1994; Brill, 1996; Dickson and Graham, 2004; Bernal et al., 2017). Pacific bluefin tuna are widely distributed across the Pacific Ocean, whereas their spawning areas are limited to Asian waters (Figure 1A). One main stock of PBT breeds in the waters of the western North Pacific Ocean between the Philippines and the Nansei Islands of Japan from April to June (Yabe et al., 1966; Chen et al., 2006), and another stock breeds in the Sea of Japan in August (Okiyama, 1974; Tanaka et al., 2007) (Figure 1A). The larvae spawned in the western North Pacific Ocean are transported by ocean currents (e.g., Kuroshio Current) to the coastal waters of Japan in the summer 2–3 months after hatching (Chen et al., 2006; Tanaka et al., 2006; 2006; Satoh et al., 2008; Kitagawa et al., 2010; Satoh, 2010) (Figure 1A). While some PBT remain in the coastal waters around Japan, others migrate from the Kuroshio–Oyashio transition region to the eastern Pacific in what is referred to as the trans-Pacific migration (Orange and Fink, 1963; Clemens and Fittner, 1969; Bayliff et al., 1991; Itoh et al., 2003; Kitagawa et al., 2009; Fujioka et al., 2018).

It has long been known that PBT juveniles with a fork length of 30 cm or more exhibit a thermal excess of 3°C–4°C post-capture compared to the ambient water (Funakoshi et al., 1985), indicating that PBT of this size and larger already have developed the endothermic ability. The long-term measurements of $T_{b}$ , $T_{a}$ , and swimming depth using biologging devices, have demonstrated that they have developed ability to maintain $T_{b}$ when more than 45 cm $L_{f}$ fish (Kitagawa et al., 2000; 2001; Kitagawa and Kimura, 2006; Kitagawa et al., 2007a; 2007b; Fujioka et al., 2018). Kubo et al. (2008) measured the ‘steady-state’ $T_{b}$ in PBT across a size range of 16.5–55.5 cm $L_{f}$ and reported that the thermal excess increased from 0.1°C to 1.5°C in individuals measuring 16.5–34.9 cm $L_{f}$ to 2.6°C–4.4°C in those measuring 54.5–55.5 cm $L_{f}$ , consistent with earlier observations in *Euthynnus* tunas (Dickson, 1994).

The mechanistic basis of thermal excess enhancement has mainly been attributed to the heat retention capacity, the development of *retia mirabilia*, because PBT develop the vascular structure rapidly during the juvenile stage (Funakoshi et al., 1985; Malik et al., 2020). Moreover, a biologging study has also shown that the heat retention capacity considerably improves with growth, while that of the heat-production rate decreases after >45 cm $L_{f}$ (Kitagawa et al., 2007b). Meanwhile, the ontogenetic progression of heat production capacity and its role in the development of their endothermy remains less comprehensively understood. Achieving a body temperature above the ambient level necessitates not only heat retention but also substantial heat production. The total heat production capacity increases with body size due to the growth of red muscle mass (Malik et al., 2020), especially during the early juvenile stage (<30 cm $L_{f}$ ) (Kubo et al., 2008), implying an ontogenetic increase in heat production capacity.

Recent technological advancements have enabled the miniaturization of biologging devices and the *in situ* measurement of body temperature in small-sized tuna (<30 cm $L_{f}$ ), providing new insights into the dynamic developmental process of PBT’s endothermic ability (Kitagawa et al., 2022). However, our previous study focused on the developmental process of heat retention capacity but did not discuss heat production capacity (Kitagawa et al., 2022). Therefore, the present study aimed to elucidate the ontogenic development of metabolic heat production capacity in PBT and its contribution to endothermic ability through reanalysis of the dataset in the previous study. In the present study, we (1) estimated the heat-production rate in juvenile PBT *via* heat-budget model and its scaling exponents in the juvenile as an index for developmental state, (2) discussed the relationship between the parameters estimated *via* heat-budget model and the contribution of heat production to the development of endothermic ability in PBT, and (3) evaluated aerobic capacity-related traits, including metabolic rate, total red muscle mass, and ventricular mass, and estimated scaling exponents of these traits.

## 2 Material and methods

### 2.1 Analysis of biologging data

#### 2.1.1 Summary of analyzed data and electronic devices

In this study, we analyzed time-series temperature data of body ( $T_{b}$ ) and ambient water ( $T_{a}$ ) from nine juvenile Pacific bluefin tuna for a heat-budget model (Table 1). The data were originally obtained from tagging survey conducted on PBT by the National Institutions of Far Seas Fisheries, Fisheries Research Agency (present name: Japan Fisheries Research and Education Agency Fisheries Resources Institute) in Tosa Bay, Kochi Prefecture (Furukawa et al., 2017; Fujioka et al., 2018; Kitagawa et al., 2022) from July to August in 2012–2015.

**TABLE 1:** Information on the individuals used for heat-budget model analysis. Fish size is expressed in fork length. The parentheses in size range analysis column indicate size estimated from growth rate due to the lack of size information at recapture.

Fish ID	Capture/Recapture date	Tagging/Recapture size (cm)	Size range for analysis (cm)	Growth rate (cm· ${\text{day}}^{-1}$ )	Data length (days)
2012-0925	July 28, 2012/August 25, 2014	24.0/n.a	24.0–(52.6)	0.24	120
2012-0932	July 29, 2012/December 19, 2012	28.0/53.5	28.9–47.1	0.18	102
2012-0948	July 29, 2012/October 21, 2012	24.5/50.0	28.9–42.8	0.18	78
2012-1,127	August 10, 2012/ July 28, 2013	26.5/n.a	26.5–(55.3)	0.24	120
2013-1766	August 15, 2013/September 19, 2014	23.0/n.a	23.0–(51.3)	0.24	120
2014-2,880	August 14, 2014/November 18, 2014	21.5/47.2	23.4–47.1	0.27	88
2014-2,922	August 18, 2014/November 13, 2014	23.0/49.0	23.0–49.0	0.30	87
2014-2,952	August 20, 2014/November 13, 2014	25.0/46.5	25.0–46.2	0.25	85
2015-3792	August 02, 2015/October 21, 2015	26.5/47.2	26.5–47.0	0.26	79

Over the 4-year tagging survey, a total of 3,281 PBT juveniles were captured by trawling in the coastal area of Tosa Bay (2012–2015: *n* = 1,044, 1,725, 236, 276), and 2,518 fish were released with dart tags (2012–2015: *n* = 923, 1,147, 201, 247). Of the dart-tagged fish, 321 fish were surgically implanted with an archival tag (LAT2910; Lotek Wireless Inc. Ontario, Canada) into their peritoneal cavity and released from the coastal area (2012–2015: *n* = 75, 62, 77, 107). In total, 307 fish were recaptured off Tosa Bay, its adjacent waters, and in California, United States (2012–2015: *n* = 128, 60, 45, 74). Of these, 93 were archival-tagged individuals (2012–2015: n = 23, 8, 23, 39), but for about half of the fish, the archival tags themselves were not recovered, or the data were not retrieved due to the tag malfunction. As a result, 41 fish were used for the heat-budget model in our previous study (Kitagawa et al., 2022). In this study, we selected nine individuals with more than 2 months of time-series data for analysis (Table 1), excluding 32 individuals with shorter data records, because this study aimed to evaluate the development of endothermic capacity from the 20 to >40 cm size range.

The archival tags consisted of a body ( ϕ8.5×25 mm) and stalk (154 mm), which weighed 3.3 g and 1.2 g in air and water, respectively. A temperature and pressure sensor were attached to the main body, and another temperature and illuminance sensor were attached to the tip of the stalk. The recorded temperatures at the main body were used as $T_{b}$ in the peritoneal cavity (where the tag was placed in) and those at the stalk as $T_{a}$ . The illuminance and pressure sensors were set to measure light level and depth, respectively. The sampling intervals of all sensors were set to 30 s (Furukawa et al., 2017; Fujioka et al., 2018; Kitagawa et al., 2022). The temperature sensors had a resolution of 0.02°C in the range of −5–45°C.

The tagging procedure was described in detail in previous studies (Furukawa et al., 2017; Fujioka et al., 2018). Briefly, a scalpel was used to make a 1 cm incision along the body approximately 0.5 cm from the midline and 1–2 cm anterior to the anus, through which the archival tag was inserted into the peritoneal cavity. At the tagging timing, the straight fork length of each fish ( $L_{f}$ , in cm) was measured. The implantation procedure was generally completed within 30 s.

#### 2.1.2 Time-series data analysis

Igor Pro Ver 8.1 (WaveMetrics Inc., Portland, OR, United States) and its add-on package of Ethographer (Sakamoto et al., 2009) were used to analyze the $T_{a}$ and $T_{b}$ , recorded at 30s intervals by the archival tags. Temporary measurement abnormalities were corrected by taking a moving average of the values before and after the abnormal point (less than 0.01% of all data points). The diurnal pattern has been known in the PBT’s body temperature (Kitagawa et al., 2022), where the temperatures in the daytime were higher than those in the nighttime, which generally reflects an increase in metabolic demand accompanied by activity and digestion in the daytime. In the present study, to reveal the developmental process of body temperature in the stable condition of early juvenile stages (20–40 cm $L_{f}$ ), the data during nighttime were focused on the analysis, assuming as a low-activity phase (10 p.m.–5 a.m.). Additionally, the data were limited to the first 120 days after release (Table 1), although our previous analysis had extended to the following year after the release (Kitagawa et al., 2022). This limitation was applied because estimating the increase in body size (fork length) during winter was difficult due to the reduced growth rate during this period.

#### 2.1.3 Heat-budget model (HBM)

To analyze body temperature dynamics in juvenile PBT, we employed a heat-budget model to estimate changes in the whole-body heat-transfer coefficient ( λ ) and heat-production rate ( ${\dot{T}}_{m}$ ) according to previous studies (Kitagawa and Kimura, 2006; Nakamura et al., 2015; Kitagawa et al., 2022). The heat-budget model follows the equation:

$$\frac{dT_{b}}{dt}\left(t\right)=\lambda \left(T_{a}\left(t\right)-T_{b}\left(t\right)\right)+{\dot{T}}_{m},$$ (1)

where, $T_{b}\left(t\right)$ represents body temperature (in °C) as a function of time ( t , in min), $T_{a}$ is ambient water temperature (in °C) as a function of t . The parameter λ indicates the whole-body heat-transfer coefficient ( $\min^{-1}$ ), which represents the rate of heat exchange between the body and ambient water. The term ${\dot{T}}_{m}$ represents the heat-production rate (°C $\cdot \min^{-1}$ ), which indicates the rate of internal heat generation. Therefore, the heat-budget model shows that the time change in body temperature at a time t ( $dT_{b}/dt\left(t\right)$ , in °C $\min^{-1}$ ) is determined by the rate of heat exchange (first term) and heat production (second term).

In our previous study (Kitagawa et al., 2022), we assumed that the ambient water temperature at a given time, $T_{a}\left(t\right)$ , reflects the time change in $T_{b}$ at the same time, $dT_{b}/dt\left(t\right)$ However, a time lag in heat transfer response was found. To account for this, the response time lag against $T_{a}\left(t\right)$ ( τ , in min) was added to Equation 1:

$$\frac{dT_{b}}{dt}\left(t\right)=\lambda \left(T_{a}\left(t-\tau \right)-T_{b}\left(t\right)\right)+{\dot{T}}_{m}.$$ (2)

The parameters were estimated for each day using maximum likelihood method. We used the “lm” function in R [v.4.3.1, R Core Team (2023)] to estimate the parameters for models with different values of τ , and calculated the Bayesian information criterion (BIC) for each model according to previous studies (Nakamura et al., 2015; 2020) The model with the lowest BIC values was regarded as the more parsimonious model, and the parameters estimated by the lowest BIC model were used as parameters for the day.

#### 2.1.4 Allometry of HBM parameters

To clarify the development of the heat-production rate in PBT, the heat-production rate was compared to body mass. The relationship between body mass ( $M_{b}$ ) and a trait ( I ) is generally described by a power-law equation (Schmidt-Nielsen, 1984), known as the allometric equation, as follows:

$$I=\alpha {M_{b}}^{\beta },$$ (3)

where, α is the scaling coefficient, and β is the scaling exponent or slope of the log-log plot of I vs $M_{b}$ (Glazier, 2005; Killen et al., 2010). The scaling exponent indicates the rate of increase of a trait relative to the increase in body mass. In this study, we evaluated the development of the heat-production rate using the allometric equation. To apply linear models, both body mass ( $M_{b}$ ) and heat-production rate ( ${\dot{T}}_{m}$ ) were $\log_{10}$ -transformed. Estimation was first conducted on a linear model using the lm function in R. To explore whether there is a change point in the allometric relationship of heat-production rate during ontogeny, segmented regression analysis was performed on the linear model, comparing the Akaike information criterion (AIC) and BIC to determine if breakpoints should be included. The segmented function in the “segmented” package of R was used for this analysis (Muggeo, 2008).

#### 2.1.5 Body size estimation of the tagged PBT

To estimate the scaling exponent of the heat-production rate, the body mass of PBT juveniles on each day was estimated based on a calculation in a previous study (Kitagawa et al., 2022). Briefly, the estimation was conducted through two processes: (1) estimating the fork length on each day using the growth rate, and (2) estimating the body mass from the estimated fork length. The growth rate of PBT’s fork length is rapid and linear in 0-age fish, for example, at 0.45 $\text{cm}\cdot {\text{day}}^{-1}$ at 30–120 days of age (Jusup et al., 2011). Even after 4 months of age, the rapid growth of 0-age fish is known to continue until the onset of their first winter (Fukuda et al., 2015b). PBT juvenile do not fit the von Bertalanffy growth function (Von Bertalanffy, 1938) otherwise widely used as a growth formula (Fukuda et al., 2015a), where the fork length during ontogeny is described by a sigmoid curve. Therefore, the increase in fork length during the analysis period (0–120 days after release) was estimated by linear regression between fork length and the days after release. For fish without fork length data at the time of recapture ( n=3 ; Table 1), fork length was estimated from the average growth rate (0.24 $\text{cm}\cdot {\text{day}}^{-1}$ ) number based on linear regression of other fish ( n=24 ) in a previous study (Kitagawa et al., 2022). Body mass ( $M_{b}$ ) during the analysis period was estimated from fork length ( $L_{f}$ ) using the allometric formula (Equation 3), $M_{b}=\alpha_{L}{L_{f}}^{\beta_{L}}$ [ $\alpha_{L}=4.85\cdot 10^{-3}$ , $\beta_{L}=3.39$ , the values of the constant from Malik et al. (2020)].

### 2.2 Metabolic rate measurement

#### 2.2.1 Fish collection and maintenance

Swimming respirometry was conducted at the Iburi Center (IC) of Osaka Kaiyukan Marine Biological Research Institute (Figure 1B) from August 9 to 26, 2022, and from August 15 to 9 September 2023. Juvenile Pacific bluefin tuna, ranging from 16.6 to 28.2 cm in fork length, were captured by hook-and-line trolling over a period of 2–3 days (August 11–13, 2022, August 18–19, 2023) off the waters of Tosa Bay, Japan. The captured fish were transported to IC on the final day of fishing each year. Upon arrival, the fish were transferred from the transport tank to 5-ton holding tanks (diameter 2.6 m, depth 0.94 m) with a custom-made dip-net, where the lower part was made of vinyl sheet and thus filled with water during fish handling. A total of 97 fish (2022: n=67 , 2023: n=30 ) were collected and 31 fish (2022: n=16 , 2023: n=15 ) were used for swimming respirometry. The holding tanks were aerated and supplied with filtered seawater. The holding tank water was maintained at the sea surface temperature at the location where the fish were caught (mean ± s.d.: 25.8°C ± 0.5°C). The PBT were fed larvae of Japanese anchovy (*Engraulis japonica*), to saturation twice daily, with the total daily feeding amounting to approximately 5%–10% of their body mass. They were not fed at least 12 h prior to the start of the swimming experiment. After the swimming experiment, the remaining fish not used for the experiment were euthanized with an overdose of FA100 (4-allyl-2-methoxyphenol, known as eugenol, 107 $\text{mg}\cdot {\text{mL}}^{-1}$ ; Tanabe Seiyaku Co. Ltd., Osaka, Japan). The euthanized PBT juveniles were dissected for sampling, including ventricular mass measurement, or whole body frozen for red muscle mass measurement. The samples were then stored in a freezer at −20°C.

#### 2.2.2 Swimming respirometry

A Steffensen-type swim tunnel respirometer (SW10210, Loligo Systems, Viborg, Denmark) situated at the IC was used to measure the oxygen consumption rate ( ${\dot{M}}_{O_{2}}$ , in $\text{mg}O_{2}\cdot h^{-1}$ ) of the fish as a function of swimming speed (Svendsen et al., 2016). The tank in the swimming section of the respirometer held 90 L of water; water flow was generated using a voltage-controlled motor and propeller, where the voltage was calibrated against the water velocity. The swim tunnel was connected to an optical O_2_ sensor instrument (Firesting O_2_; PyroScience GmbH, Aachen, Germany) using the dipping probe oxygen minisensor included in the instrument. The external water bath of the swim tunnel was connected to a plastic supply tank containing 200 L of air-saturated water. The water temperature of the supply tank was maintained in 25.6°C–26.4°C during the ${\dot{M}}_{O_{2}}$ measurement. The respirometer was darkened on the outside using a black curtain to prevent external disturbances.

The fish were transferred from the holding tank to the swim tunnel using a nylon sling. The fish were first given 0.5–3 h to acclimate to the swim tunnel at a water speed of 45–60 $\text{cm}\cdot s^{-1}$ (approx. 1.5–2.1 $L_{f}\cdot s^{-1}$ ), at which speed they swim regularly. Conversely, under a certain low flow speed (<25–35 $\text{cm}\cdot s^{-1}$ ), they could not maintain their posture horizontally and opened their mouths, and eventually started to show active ventilation, resulting in increased ${\dot{M}}_{O_{2}}$ compared to ${\dot{M}}_{O_{2}}$ at a speed of 45–60 $\text{cm}\cdot s^{-1}$ . Some fish failed to maintain position in the swim tunnel, or repeatedly charged the upstream screen of the swim tunnel by 1 h after placing them in the swim chamber. In such a case, the fish was immediately removed from the swim tunnel and a new trial with a new fish started. During the acclimation period, the oxygen consumption rate was monitored, and confirmed that the ${\dot{M}}_{O_{2}}$ for all individuals were almost stabilized (defined by Iino et al. (2024) as an estimated ${\dot{M}}_{O_{2}}$ that did not vary by more than 10% from the mean over three consecutive measurement cycles). Each experimental run consisted of a 3–8 min period at each of the designated speeds up to 90 $\text{cm}\cdot s^{-1}$ . After each measurement, the seawater was exchanged with fresh seawater.

After each 15 min period, the water flow was increased by an additional 0.3 $L_{f}\cdot s^{-1}$ and was maintained at the new velocity for 15 min or until the fish were unable to swim against the current and were pushed to the downstream screen, and remained there for more than 10 s. After each trial, the fish were euthanized by overdose of anesthesia (FA100), and the fork length and body mass were measured. Some fish were dissected soon after euthanasia for sampling, while others were frozen whole bodies for later quantification of red muscle mass.

The oxygen consumption rate ( ${\dot{M}}_{O_{2}}$ , in ${\text{mgO}}_{2}\cdot \min^{-1}$ ) was determined during the measurement period ( Δt , in min ) as the decline in dissloved oxygen ( $\Delta C_{O_{2}}$ , in ${\text{mgO}}_{2}$ ) in the swim tunnel. ${\dot{M}}_{O_{2}}$ was calculated as:

$${\dot{M}}_{O_{2}}=\frac{\Delta C_{O_{2}}}{\Delta t}\left(V_{ch}-V_{b}\right),$$ (4)

where $V_{ch}$ is the volume of the swim chamber (in l), $V_{b}$ is the volume of the fish (in l, which was calculated from body mass assuming the density of the fish was 1 $\text{kg}\cdot l^{–1}$ ). Background changes in dissolved oxygen concentration, measured when there were no fish in the swim chamber, were negligible. The swim speed was corrected for blocking effects, as recommended by Bell and Terhune (1970), when the cross-sectional area of the fish exceeded 2% of the swimming chamber area (Iino et al., 2024).

#### 2.2.3 Scaling of metabolic rate

In this study, the standard metabolic rate (SMR) was determined to calculate the scaling exponent of a metabolic trait. The SMR is defined as the metabolic rate when swimming speed is zero, and for tunas, it is typically derived from the relationship between metabolic rate and swimming speed, known as the “swimming curve” (Dewar and Graham, 1994; Sepulveda and Dickson, 2000). Previous studies have reported a linear relationship between metabolic rate and swimming speed in tunas, and this study also identified a similar linear relationship (Dewar and Graham, 1994; Sepulveda and Dickson, 2000). Consequently, a linear model was employed for the estimation, where the oxygen consumption rate at a given speed ( ${\dot{M}}_{O_{2}}\left(U\right)$ ) can be expressed as follows:

$${\dot{M}}_{O_{2}}\left(U\right)=\gamma U+{\dot{M}}_{O_{2},\text{SMR}},$$ (5)

where U denotes swimming speed ( $\text{cm}\cdot s^{-1}$ , and γ represents the slope of the linear function. The intercept indicates the oxygen consumption rate when the swimming speed is zero, thus providing an estimate of the SMR. For each individual, the swimming curve was estimated using a linear model, and the metabolic rate at a swimming speed of zero was defined as the SMR. However, since the metabolic rate estimated from the swimming curve is obtained through extrapolation, some concerns have been raised regarding the accuracy of such estimates (Chabot et al., 2016). To address this issue, we also determined the metabolic rate at the minimum (sustained) swimming speed ( ${\dot{M}}_{O_{2},U_{\min}}$ ), defined as the lowest speed at which tunas can maintain their swimming, and used ${\dot{M}}_{O_{2},U_{\min}}$ for the metabolic trait scaling exponent calculation.

The minimum swimming speed ( $U_{\min}$ ) was determined for six individuals in 2023 by gradually reducing the flow speed after the measurement phase. Specifically, after the measurements, the flow speed was decreased incrementally at a speed of 5 $\text{cm}\cdot s^{-1}$ , and the $U_{\min}$ was defined as the speed just before the metabolic rate began to increase. Within the size range examined in this study, no significant correlation was observed between the $U_{\min}$ and body mass, so the average $U_{\min}$ of 44.3 $\text{cm}\cdot s^{-1}$ was used. By substituting the $U_{\min}$ into the swimming curve for each individual, we calculated ${\dot{M}}_{O_{2},U_{\min}}$ .

#### 2.2.4 Calculating red muscle and ventricle masses

Metabolic heat produced through aerobic metabolism in red muscles (RM) is a major source of body temperature, and the ventricle is closely related to aerobic capacity (Graham and Dickson, 2001). To evaluate the development of red muscle and ventricle in the early juvenile stage, a portion of PBT juveniles captured for swimming respirometry were measured for the masses of red muscle ( $M_{RM}$ ) and ventricle ( $M_{v}$ ).

Twenty-one fish (mean ± s.d. fork length: 20.6 ± 3.2 cm, body mass: 142.4 ± 80.6 g) were used to quantify total red muscle mass ( $M_{RM}$ , in g). The method was based on a previously described protocol (Bernal et al., 2003; Malik et al., 2020). For each fish, the total red muscle mass was calculated as the sum of the mass in the cross-sections. Before analysis, the fish were stored in −20 °C freezer and they were sectioned using a bandsaw while still frozen. Measurable quantities of RM did not occur in the anterior 20%–25% $L_{f}$ . Thus, beginning at this position, whole frozen PBT juveniles were cut into 0.6–1.4-cm thick (approx. 4%–6% $L_{f}$ ) cross-section until 70%–90% $L_{f}$ , where little to no red muscle remained visible. The anterior sides of each cross-section were photographed alongside a scale bar. For each of the cross-sections, the red muscle’s cross-sectional area was measured using ImageJ. The cross-sectional area was multiplied by the thickness of section (0.6–1.4 cm) to give the volume value, and it was then multiplied by the published density [1.05 $g\cdot {\text{cm}}^{-3}$ ; Bernal et al. (2003)] for the tuna muscle to give the mass value.

The ventricular masses ( $M_{v}$ , in g) were measured in 37 juveniles (mean ± s.d. fork length: 22.7 ± 3.4 cm, body mass: 207.0 ± 107.0 g). Each fish was dissected soon after euthanasia by anesthesia overdose. The ventricles were blotted and weighed to assess the ventricular mass. For each fish, the ventricular mass was divided by the body mass to give relative ventricular mass.

## 3 Results

### 3.1 Heat-production rate

**FIGURE 2:**
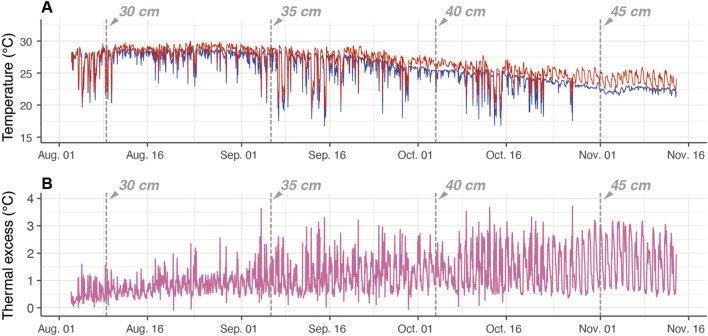
Example of time-series data of electronically tagged Pacific bluefin tuna (ID 2012-0932). The vertical dashed lines depict the estimated fork length at the time. **(A)** Body temperature ( $T_{b}$ , red) and ambient water temperature ( $T_{a}$ , blue). **(B)** The temperature difference between body and water (thermal excess).

Time-series data of the $T_{b}$ , $T_{a}$ , and $T_{X}$ retrieved from one individual (ID 2012-0932) are shown by a representative example (Figure 2). During this period, it is estimated that its fork length increased from 29 to 47 cm (approx. 500 g to 2 kg; Table 1; Figure 2). The $T_{X}$ exhibited a diurnal pattern; the daytime $T_{X}$ was higher than the nighttime $T_{X}$ . At small sizes (<30 cm $L_{f}$ ), the $T_{X}$ ranged from 0.25°C to 1.5°C, but the $T_{X}$ increased with growth, and at larger sizes (>45 cm $L_{f}$ ), the $T_{X}$ range increased to 0.5°C–3°C (Figure 2).

**FIGURE 3:**
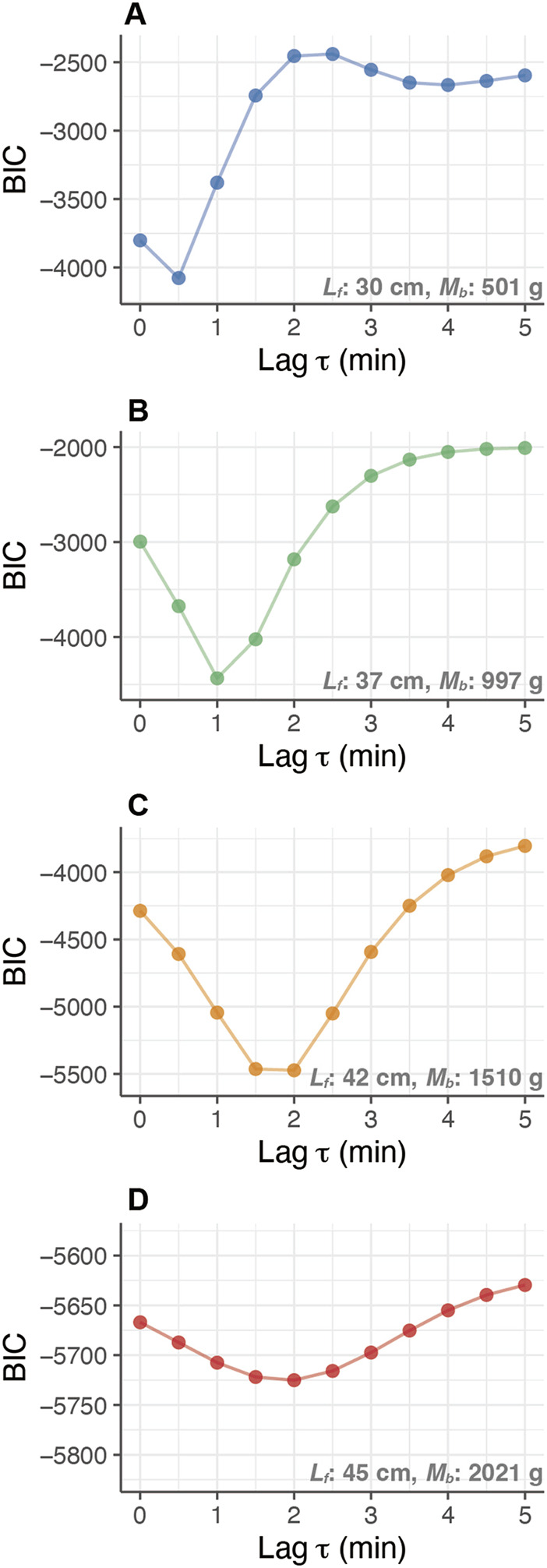
Relationship between the response time-lag (Lag τ in minutes) and the Bayesian Information Criterion (BIC) of heat-budget models with growth of PBT. The comparison of BIC values for different models with different time-lags was conducted for an individual (ID 2012-0932) at different body masses: **(A)** 0.5 kg (30 cm $L_{f}$ ), **(B)** 1.0 kg (37 cm $L_{f}$ ), **(C)** 1.5 kg (42 cm $L_{f}$ ), and **(D)** 2.0 kg (45 cm $L_{f}$ ).

For the fish, the heat-production rate ( ${\dot{T}}_{m}$ ) and whole-body heat-transfer coefficient ( λ ) were estimated using the heat-budget model (Equation 2). The response time lag τ minimizing BIC of the heat-budget models on each day increased, accompanied by their growth. The minimizing τ ranged 0.5–1 min at the length of 30–35 cm $L_{f}$ (approx. 500–1,000 g; Figures 3A,B), while the heat-budget model incorporating a τ of more than 2 min showed the lowest BIC over 40 cm $L_{f}$ (approx. > 1.5 kg; Figures 3C,D). The calculated BIC values of the heat-budget models were at their minimum at a response time lag ( τ ) 0–3 min in the fork length range of 25–50 cm.

**FIGURE 4:**
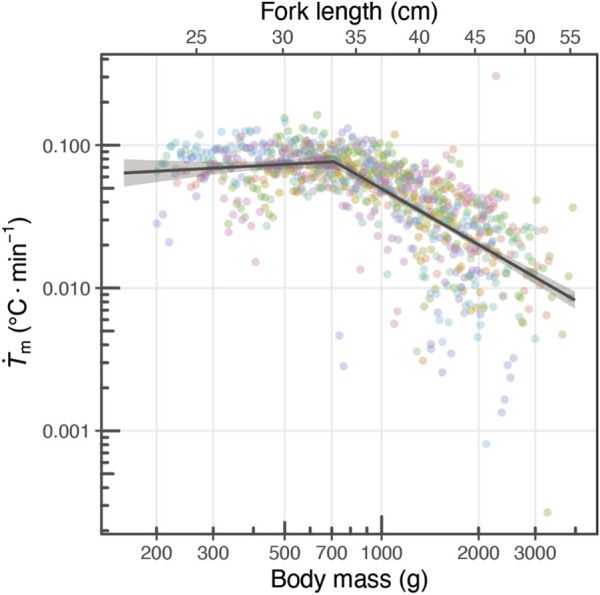
Changing relationship of ${\dot{T}}_{m}$ with their growth. The relationship between body mass and ${\dot{T}}_{m}$ is depicted, where the circles represent parameters estimated by the heat-budget model for each individual for each day. The colors of the circles correspond to different individuals. The dark gray line and shaded area represent the segment regression line and its 95% confidence interval area.

The heat-production rate ( ${\dot{T}}_{m}$ ) was estimated for individuals with fork lengths ranging from 23.0 to 55.3 cm (body weight 200–3,900 g) (Figure 4). A decreasing trend in ${\dot{T}}_{m}$ was observed with body size, but when ${\dot{T}}_{m}$ was plotted against body weight on both logarithms, ${\dot{T}}_{m}$ tended to remain constant up to a certain body mass (Figure 4). The segmented regression model exhibited lower AIC and BIC values than the linear model (segmented model: AIC=85.1 ; BIC=108.9 , linear model: AIC=153.5 ; BIC=167.7 ), which indicated a change in the slope of ${\dot{T}}_{m}$ at the breakpoint (714 g, 95%CI: 641.7–794.5).The slope of ${\dot{T}}_{m}$ remained close to 0 before the breakpoint (i.e., below 714 g), with no significant slope detected (estimate: 0.121 , 95%CI: −0.082 – 0.324 , p=0.74 ) (Figure 4). In contrast, beyond the breakpoint (i.e., above 714 g), ${\dot{T}}_{m}$ decreased with increasing body weight (Estimate: −1.299 , 95%CI: −1.421 – −1.176 , p<0.01 ).

### 3.2 Relationship between the development of heat-production rate and endothermic ability

In the case of $T_{b}$ is an equilibrium state—meaning the state where the left side of the heat-budget model is 0 (Equation 1)—the temperature difference between body and ambient water ( $T_{b}-T_{a}$ ), or thermal excess ( $T_{X}$ ), can be described by the following:

$${\dot{T}}_{m}=\lambda T_{X},$$ (6)

where the $T_{X}$ is determined by the values of ${\dot{T}}_{m}$ and λ . For example, if ${\dot{T}}_{m}$ is twice λ , $T_{X}$ is calculated as 2°C. Furthermore, if this equation is converted into logarithmic form, Equation 6 becomes:

$$\log{\dot{T}}_{m}=\log\lambda +\log T_{X}.$$ (7)

**FIGURE 5:**
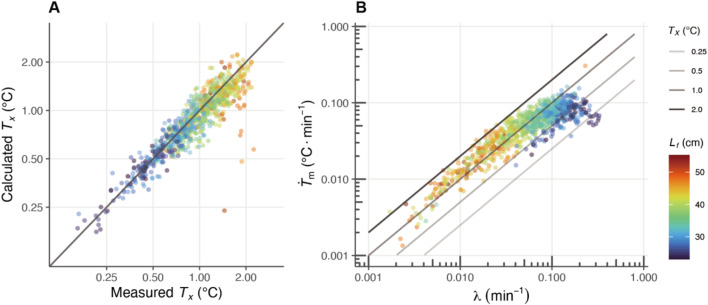
The effects of heat-production rate ( ${\dot{T}}_{m}$ ) and whole-body heat-transfer coefficient ( λ ) on the difference between body and ambient (water) temperatures (thermal excess: $T_{X}=T_{b}-T_{a}$ , °C). **(A)** The comparison of the estimated $T_{X}$ with the measured $T_{X}$ . The estimated $T_{X}$ indicates the $T_{X}$ calculated by ${\dot{T}}_{m}$ and λ, and the measured $T_{X}$ shows the mean thermal excess on each day. **(B)** The relationships between ${\dot{T}}_{m}$ and λ are shown on a log-log plot, the $T_{X}$ is expressed as the intercept. Each circular marker represents an individual fish, and the marker colors correspond to the fork length ( $L_{f}$ , in cm), as indicated by the color bar on the right. The solid lines represent different temperature differentials between body and water temperatures ( $T_{X}$ ), with thicker lines indicating larger differentials.

In this equation, the relationship between $\log{\dot{T}}_{m}$ and log⁡λ is expressed as a linear function with slope 1 and the $\log T_{X}$ as the intercept of the linear function. The estimated $T_{X}$ using the HBM parameters was consistent with the mean thermal excess on each day, calculated as the difference between the mean $T_{b}$ and the mean $T_{a}$ (Figure 5A).

The HBM parameters estimated for each day were plotted on a log-log graph, revealing that the thermal excess increased as λ decreases with growth (Figure 5B). Focusing on the fork length range of 25–35 cm, thermal excess rised from 0.25°C to 1°C (Figure 5), and within this range, the distribution of the plots shifted horizontally from right to left (Figure 5B). The moving pattern of the plots suggests that, in addition to the decrease in λ , the maintenance of a high ${\dot{T}}_{m}$ is essential for the increase in thermal excess between 25 and 35 cm (Figure 5B). In contrast, beyond this size range, although the values of ${\dot{T}}_{m}$ decrease, thermal excess continues to increase due to the more pronounced decline in λ (Figure 5B).

### 3.3 Swimming respirometry

**FIGURE 6:**
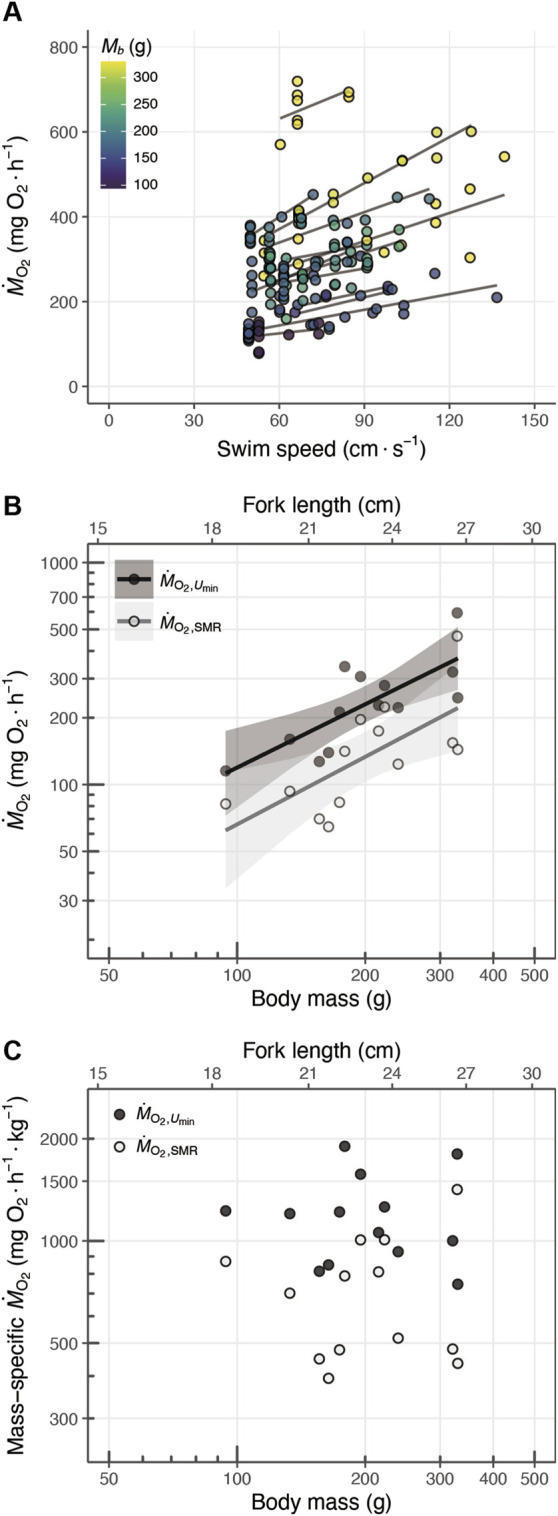
**(A)** Relationship between the swim speed ( $\text{cm}\cdot s^{-1}$ ) and oxygen consumption rate ( $\text{mg}O_{2}\cdot h^{-1}$ ), where point color represents body mass ( $M_{b}$ , in g). The solid lines represent the fitting results for each individual fish. **(B)** Scaling of and standard metabolic rate ( ${\dot{M}}_{O_{2},\text{SMR}}$ , pale color; scaling exponent: 0.95) and metabolic rate at minimum swim speed ( ${\dot{M}}_{O_{2},U_{\min}}$ , dark color; scaling exponent: 1.01) with body mass. **(C)** Scaling of and mass-specific standard metabolic rate ( ${\dot{M}}_{O_{2},\text{SMR}}$ , pale color) and mass-specific metabolic rate at minimum swim speed ( ${\dot{M}}_{O_{2},U_{\min}}$ , dark color) with body mass. The solid lines indicate the linear regression fits, and the shaded areas show the 95% confidence intervals.

The oxygen consumption rate was linearly correlated with the swim speed (Figure 6A) (Equation 5). The minimum swim speed was evaluated for six fish (mean ± s.d. fork length: 23.0 ± 3.5 cm, body mass: 193 ± 90 g) by decreasing the flow speed. The average speed was 44.3±1.51 (±s.d.) $\text{cm}\cdot s^{-1}$ . The minimum swim speed did not significantly correlate with body mass within the size range ( p=0.62 ). Therefore, the minimum swim speed in the ${\dot{M}}_{O_{2},U_{\min}}$ estimate was set to the average of 44.3 $\text{cm}\cdot s^{-1}$ .

${\dot{M}}_{O_{2},U_{\min}}$ and ${\dot{M}}_{O_{2},\text{SMR}}$ ranged 115.2–593.3 $\text{mg}O_{2}\cdot h^{-1}$ and 64.6–466.3 $\text{mg}O_{2}\cdot h^{-1}$ , respectively (Figure 6B). The both of ${\dot{M}}_{O_{2},U_{\min}}$ and ${\dot{M}}_{O_{2},\text{SMR}}$ increased with body mass (Figure 6B). The scaling exponent of ${\dot{M}}_{O_{2},U_{\min}}$ was 0.95 (s.e.: 0.24) and that of ${\dot{M}}_{O_{2},\text{SMR}}$ was 1.01 (s.e.: 0.33) (Table 3).

The mean (±s.d.) value of mass-specific ${\dot{M}}_{O_{2},U_{\min}}$ was 719.7 (±303.5) $\text{mg}O_{2}\cdot h^{-1}$ and that of mass-specific ${\dot{M}}_{O_{2},\text{SMR}}$ was 1,198.3 (±366.1) $\text{mg}O_{2}\cdot h^{-1}$ (Table 2; Figure 6C). The scaling exponent of the mass-specific ${\dot{M}}_{O_{2},U_{\min}}$ was −0.05 (s.e.: 0.24) and that of the mass-specific ${\dot{M}}_{O_{2},\text{SMR}}$ was 0.33 (s.e. 0.33), but both exponents were not significantly different from zero (Table 3).

**TABLE 2:** Summarized information on fish body size and measurements of physiological traits. Each physiological trait is represented as a value relative to body mass. Mean ± s.d., and min-max range (in parentheses) are presented.

Trait	*n*	Fork length (cm)	Body mass (g)	Relative values to body mass	Units
${\dot{M}}_{O_{2},\text{SMR}}$	13	23.3 ± 2.5 (19.4–27.5)	211.6 ± 75.7 (94.0–330.0)	719.7 ± 303.5 (393.6–1,417.4)	mg O_2_·h^−1^·kg^−1^
${\dot{M}}_{O_{2},U_{\min}}$	13	23.3 ± 2.5 (19.4–27.5)	211.6 ± 75.7 (94.0–330.0)	1,198.3 ± 366.1 (745.4–1900.0)	mg O_2_·h^−1^·kg^−1^
Red muscle	21	20.6 ± 3.2 (16.4–27.9)	142.4 ± 80.6 (48.0–362.0)	6.07 ± 1.79 (3.77–10.69)	%
Ventricle	39	22.4 ± 3.6 (16.2–28.2)	199.0 ± 109.4 (53.0–423.0)	0.21 ± 0.06 (0.08–0.48)	%

**TABLE 3:** Scaling exponents for physiological traits. Each row represents a physiological trait and its corresponding scaling exponent values, including absolute values and values relative to body mass (or mass-specific, ms). Scaling exponent values with a p<0.05 are highlighted in bold.

Abs./Rel	Trait	Units of trait	Scaling exponent estimate	*P*
Absolute	${\dot{M}}_{O_{2},U_{\min}}$	mg O_2_·h^−1^	**0.95 (0.42–1.48)**	**0.002**
	${\dot{M}}_{O_{2},\text{SMR}}$	mg O_2_·h^−1^	**1.01 (0.29–1.73)**	**0.011**
	Red muscle	g	**1.12 (0.96–1.43)**	**< 0.001**
	Ventricular mass	g	**1.15 (1.04–1.26)**	**< 0.001**
Relative to *M*_*b*_	ms- ${\dot{M}}_{O_{2},U_{\min}}$	mg O_2_·h^−1^·kg^−1^	−0.05 (−0.58–0.475)	0.831
	ms- ${\dot{M}}_{O_{2},\text{SMR}}$	mg O_2_·h^−1^·kg^−1^	0.01 (−0.71–0.73)	0.974
	Rel. red muscle mass	%	0.45 (−0.09–1.00)	0.096
	Rel. ventricular mass	%	**0.15 (0.04–0.26)**	**0.009**

### 3.4 Development of red muscle and ventricular masses

**FIGURE 7:**
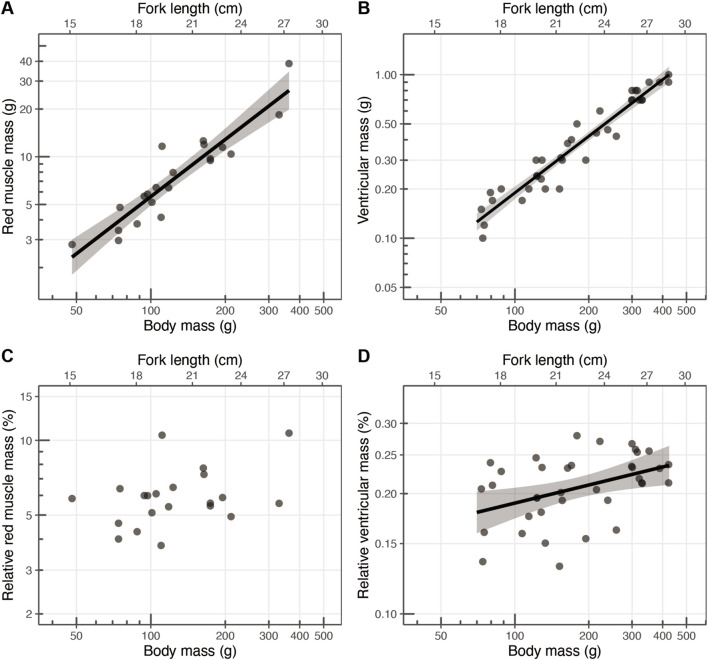
Scaling relationships between body mass (g) and **(A)** red muscle mass (g; scaling exponent: 1.12), **(B)** ventricular mass (g; scaling exponent: 1.15), **(C)** relative red muscle mass (%), and **(D)** relative ventricular mass (%; scaling exponent: 0.15). Each point represents an individual, with the solid black lines indicating the linear regression fits, and the shaded areas representing the 95% confidence intervals.

Total red muscle mass was evaluated using the fish’s cross-sectional area [ n=21 , mean ± s.d. (min–max range) fork length: 20.6 ± 3.2 (16.3–27.9) cm, body mass: 142.4 ± 80.6 (48–362) g]. The red muscle mass increased with body mass (Figure 7A) and it spanned 2.8–38.7 g in 48–362 g fish. The red muscle mass significantly increased with body mass in the log-log transformed regression model, and the scaling exponent was estimated at 1.20 (s.e.: 0.11) (Figure 7A). The ventricular mass was measured in 37 individuals [mean ± s.d. (min–max range) fork length: 22.4 ± 3.4 (17.1–28.2) cm; body mass: 199.0 ± 109.4 (73–423) g]. The ventricular masses significantly increased with body mass in the log-log transformed regression model, and the scaling exponent was estimated as 1.15 (s.e.: 0.05) (Figure 7B).

The relative value of red muscle mass to body mass ranged 3.77%–10.69% (mean ± s.d.: 6.07% ± 1.79%) (Table 2; Figure 7C), but the scaling exponent of the relative red muscle mass was not significantly larger than 0 ( p=0.09 ; Table 2). The relative ventricular mass ranged 0.132%–0.279% (Table 2; Figure 7D). The scaling exponent of the relative ventricular mass was 0.15 (s.e.: 0.054), which was significantly larger than 0 ( p=0.009 ; Table 2).

## 4 Discussion

Endothermic fish, such as tuna and lamnid sharks, can maintain the temperatures of certain tissues/organs higher than the surrounding water, if in cold water, by retaining high levels of heat production. In the early juvenile stage, a strong correlation has been observed between red muscle mass and thermal excess (Dickson et al., 2000; Kubo et al., 2008), suggesting that heat production plays an important role in thermal excess. However, thermal excess is influenced not only by heat production, but also by heat retention capacity. Since the development of the *rete mirabile* occurs around the same time as the red muscle (Funakoshi et al., 1985; Malik et al., 2020), the ontogenetic pattern of heat production capacity and the extent to which heat production specifically contributes to thermal excess remain insufficiently understood compared to the heat retention capacity. Therefore, in this study, we aimed to explore the ontogenetic pattern of heat production capacity and to discuss the extent to which heat production contributes to the thermal excess between the inside and outside of the body. The thermal excess of juvenile PBT increased with growth as shown in our previous study [Figure 2; Kitagawa et al. (2022)]. By estimating parameters using a heat-budget model, we found that heat production is maintained at a high level in the early juvenile stage. Through comparison of the parameters of the heat-budget model, we found that a high heat-production rate is important for the early formation of thermal excess. To reinforce the mechanistic basis for highly maintained heat production in early juveniles, scaling exponents were estimated for physiological and/or morphological traits related to aerobic metabolic capacity, such as metabolic rate, red muscle mass, and ventricular mass.

### 4.1 Ontogenetic patterns of heat production in PBT

The heat production rate ( ${\dot{T}}_{m}$ ) estimated by the heat-budget model decreases with growth for fish after 45 cm $L_{f}$ (Kitagawa et al., 2007b), which is natural given the scaling law of metabolic rate. Since heat production depends on metabolic heat, the internal heat production is closely related to the metabolic rate (Carey and Teal, 1969; Dewar and Graham, 1994; Blank et al., 2007a; 2007b). The heat-production rate ( ${\dot{T}}_{m}$ , in °C $\cdot \min^{-1}$ ) reflects the heating rate for a fish body (in °C $\min^{-1}\cdot {\text{ind}.}^{-1}$ ). Therefore, ${\dot{T}}_{m}$ can be interpreted as the mass-specific heating rate (i.e., °C $\min^{-1}\cdot {\text{kg}}^{-1}$ ) and the ontogenetic trend is expected to be similar to the mass-specific metabolic rate (in $\text{mg}O_{2}\cdot h^{-1}\cdot {\text{kg}}^{-1}$ ). Metabolic rate (in $\text{mg}O_{2}\cdot h^{-1}$ ) is proportional to body mass to the power of 0.5–0.9 according to the scaling laws of metabolic rate (Glazier, 2005; Killen et al., 2010), which means that the mass-specific metabolic rate (in $\text{mg}O_{2}\cdot h^{-1}\cdot {\text{kg}}^{-1}$ ) decreases in proportion to the −0.5 to the −0.1 power of body mass. The scaling exponent of metabolic rate throughout life history depends on the species, and species with higher metabolic rates exhibit lower scaling exponents (Killen et al., 2010). The scaling exponents of metabolic rates in tuna species estimated using >40 cm $L_{f}$ fish ranged from 0.5 to 0.6 (Killen et al., 2010), which is consistent with the decline in ${\dot{T}}_{m}$ with growth in PBT larger than a certain size (>45 cm $L_{f}$ in Kitagawa et al. (2007b); > approx. 35 cm $L_{f}$ in Figure 4). In contrast to the decline in ${\dot{T}}_{m}$ after 45 cm $L_{f}$ (Kitagawa et al., 2007b), in the present study, we observed that ${\dot{T}}_{m}$ did not decrease in the early juvenile stages (approx. < 35 cm, Figure 4). These results might seem to contradict the scaling laws; however, it is known that the allometry of traits, particularly metabolic rate, is not constant throughout life history but varies depending on the life history stage (Glazier, 2005; Killen et al., 2010). Previous studies have reported that the scaling exponent of the metabolic rate tends to be higher during early life stages in fish (Glazier, 2005).

### 4.2 Contribution of juvenile-specific high heat production into endothermic ability in juvenile PBT

We aimed to discuss the extent to which heat production contributes to the rise in thermal excess by comparing parameters estimated using a heat-budget model (Figure 5). The heat-budget model estimates parameters for heat production and heat retention capacity, denoted as ${\dot{T}}_{m}$ and λ , respectively. Assuming the body temperature is in a steady state, the ratio of these parameters is considered to determine the thermal excess ( $T_{X}$ ) (Equations 6 and 7). The thermal excess in juvenile PBT reaches approximately 2°C by the time they grow to approximately 45 cm $L_{f}$ (Figure 5A), which corresponds with the average nighttime body temperature. The distribution of plots before reaching 45 cm $L_{f}$ shifts to the left between 25 and 35 cm $L_{f}$ , indicating an increase in thermal excess from approximately 0.25°C–1°C (Figure 5B). Based on the pattern of this shift, it can be inferred that, as suggested by previous studies (Kitagawa et al., 2007b; Kitagawa et al., 2022), the decrease in λ plays an important role in the increase in thermal excess. Additionally, it was found that the maintenance of high heat production levels also contributed to the rapid increase in thermal excess during the early juvenile stage. Although heat production began to decline after 35 cm $L_{f}$ , it is likely that the improvement in heat retention capacity exceeded the rate of decrease in heat production, resulting in a continued enhancement in thermal excess (Figure 5B). One possible ecological reason that prevents the increase of the thermal excess due to high heat production from occurring after 35 cm $L_{f}$ is the energetic cost. Tunas require an enormous amount of food to maintain their elevated body temperatures (Estess et al., 2014). According to model estimates, the cost of heat production is substantial, with more than 80% of assimilated energy being lost after reaching 35 cm $L_{f}$ (Jusup and Matsuda, 2015), suggesting that reliance on high heat production to maintain body temperature may impose excessive energy costs.

Figure 5B also provides insights into the challenges small-sized fish face in maintaining thermal excess. For example, the plots of ${\dot{T}}_{m}$ and λ for individuals smaller than 25 cm $L_{f}$ were mainly distributed 0.05°C–0.1°C · min −1 and 0.2–0.4 min −1 , respectively. Juvenile PBT of this size exhibited a $T_{X}$ of 0.25°C; however, assuming that the $T_{X}$ increase up to 1°C, the fish would require a 2- to 8-fold increase in ${\dot{T}}_{m}$ (i.e., 0.2°C–0.4°C · min −1 ). Meanwhile, the juveniles around 40 cm $L_{f}$ showed a $T_{X}$ of 1°C, with the ${\dot{T}}_{m}$ of 0.04°C–0.1°C · min −1 and the λ of 0.05–0.1 min −1 , and a 1- to 5-fold ${\dot{T}}_{m}$ (i.e., 0.1°C–0.2°C · min −1 ) would be needed to increase $T_{X}$ to 2°C. Therefore, it is considered difficult for the small-sized fish to produce a $T_{X}$ of more than 1°C due to low heat retention ability, which also implies the limitation of body size to produce a certain level of $T_{X}$ .

### 4.3 Mechanistic basis of juvenile-specific high heat production

To examine the juvenile-stage-specific development of aerobic capacity, metabolic rate, red muscle mass, and ventricular mass, we measured and evaluated their scaling exponents (Table 3). The mass-specific ${\dot{M}}_{O_{2},\text{SMR}}$ and ${\dot{M}}_{O_{2},U_{\min}}$ of PBT juveniles were 719.7 ± 303.5 $\text{mg}O_{2}\cdot h^{-1}\cdot {\text{kg}}^{-1}$ and 1,198.3 ± 366.1 $\text{mg}O_{2}\cdot h^{-1}\cdot {\text{kg}}^{-1}$ (mean ± s.d.), respectively (Table 2; Figure 6). The mass-specific SMR of PBT was significantly higher than that of other ectothermic fish species. Although measurements of the early juvenile stages of tuna are limited, kawakawa tuna (*E. affinis*) is the only species for which a swimming curve has been provided at 24°C (Sepulveda and Dickson, 2000). The mean ± s.d. of mass-specific ${\dot{M}}_{O_{2},\text{SMR}}$ and the mass-specific ${\dot{M}}_{O_{2},U_{\min}}$ (calculated at 44.3 $\text{cm}\cdot s^{-1}$ ) of kawakawa tuna ( n=8 , $L_{f}$ range: 18.2–25.5 cm, $M_{b}$ range: 59–265 g) derived from the swimming curve were 616.9 ± 289.9 $\text{mg}O_{2}\cdot h^{-1}\cdot {\text{kg}}^{-1}$ and 1,052.5 ± 257.5 $\text{mg}O_{2}\cdot h^{-1}\cdot {\text{kg}}^{-1}$ , respectively (Sepulveda and Dickson, 2000), which were generally consistent with the values estimated in this study. The ventricle, which is an organ critical for blood circulation, had a relative mass of 0.21% ± 0.04% (mean ± s.d.) (Table 2). The relative ventricular mass of tunas ( $M_{b}$ range: 280 g–37 kg) ranges from 0.2% to 0.4% (Poupa et al., 1981; Brill and Bushnell, 1991; Graham and Dickson, 2001; Blank et al., 2004), in agreement with our results and notably higher than that of ectothermic fish species (2–2000 g), which ranges from 0.07% to 0.17% (Driedzic and Stewart, 1982; Santer et al., 1983; Sidell and Driedzic, 1985; Farrell et al., 1988). Additionally, the proportion of red muscle mass was 6.07% ± 1.79% (mean ± s.d.) (Table 2), which was also higher than that of other ectothermic fish species (Graham and Dickson, 2001).

In the early juvenile stage (15–35 cm $L_{f}$ ), the scaling exponents of these physiological traits were close to or greater than 1.0, and those of the mass-specific values (or values relative to body mass) were also close to or greater than 0 (Table 3), indicating that the traits increased isometrically or more. Although the scaling exponents of metabolic rate have not been reported not only for PBT but also for other bluefin tuna species, as it is known that scaling exponents tend to be similar among species that are phylogenetically related and have a comparative lifestyle (Killen et al., 2010). Therefore, it is reasonable to expect that bluefin tuna would have similar scaling exponents to other tuna species. The scaling exponents of other tuna species ranged from 0.5 to 0.6 [e.g., *Thynnus albacares*: 0.57, *Katsuwonus pelamis*: 0.56, *E. affinis*: 0.50, as reported by Killen et al. (2010)]. The scaling exponents for both ${\dot{M}}_{O_{2},\text{SMR}}$ and ${\dot{M}}_{O_{2},U_{\min}}$ were close to 1.0, and tended to be high compared to the previous study. The confidence intervals for the scaling exponents in ${\dot{M}}_{O_{2},\text{SMR}}$ and ${\dot{M}}_{O_{2},U_{\min}}$ were wider because of the large variations in metabolic rates between individuals and the narrow size range measured. Because metabolic rates are generally known to vary 2- to 3-fold among individuals (Burton et al., 2011), it is necessary not only to increase the sample size but also to measure a wider range of sizes in order to more accurately determine and discuss the juvenile-specific scaling exponents; however, the scaling exponents of red muscle mass and ventricular mass would corroborate the juvenile-specific scaling exponent of metabolic traits.

For red muscle, it has been reported that the scaling exponent for PBT 20–60 cm $L_{f}$ was 0.9, and the relative mass of red muscle to body decreased from approximately 7%–4% within this size range (Malik et al., 2020). In the 15–30 cm $L_{f}$ range, the scaling exponent was close to 1.0, suggesting that in this size range, the red muscle is expected to grow isometrically and to decrease after this size range. Little is known about the scaling of ventricular mass in tunas, but the ventricular mass is expected to increase beyond the 30 cm $L_{f}$ as the scaling exponent exceeded 1.0, and the relative ventricular mass also increased in this study (Table 3). Although it is not known to what extent the PBT’s ventricle relatively increases, the relative ventricular mass has been reported to be 0.32% in approximately 65 cm $L_{f}$ fish (6.0–7.5 kg) (Blank et al., 2004). This study observed that the high scaling exponents in the ventricular mass, but the interpretation of ventricular mass and its high scaling exponent should be noted. The ventricle is composed of two layers, an inner spongy layer and an outer dense layer (Poupa and Lindström, 1983). The dense layer is called the compact layer, and the compact layer plays an important role in the pumping of blood. The high scaling exponents could be attributed to an increased ratio of the compact layer to the spongy layer, not simply an increase in ventricular volume. Further histological study is required for the interpretation of the high scaling exponent in ventricular mass. In addition, the development of other tissues/organs could also contribute to that of endothermic ability. For example, the study on bigeye tuna (*Thunnus obesus*) showed the importance of white muscle on thermogenesis and thermoconservation ability (Boye et al., 2009). The present study observed that the high scaling exponents for certain physiological traits—metabolic rate, red muscle mass, and ventricular mass—during the early juvenile stages in PBT, which supports the ontogenetic pattern of heat production capacity at this stage, although other traits could also contribute to the development of endothermic ability. These results align with recent findings of higher scaling exponents specific to early life history and the hypothesis that these patterns can be attributed to relative increases in tissues with higher oxygen demand (Oikawa and Itazawa, 1993; Glazier, 2005).

### 4.4 Ecological implications of high metabolic rate

Although high heat production should be associated with high energetic costs, the ontogenetic pattern of ${\dot{T}}_{m}$ suggest that juvenile PBT maintains a high heat-production rate during the early juvenile stage, contributing to an increase in thermal excess. The elevated body temperature of tunas is thought to allow them to maintain a high metabolic rate, conferring advantages for growth and swimming performance (Brill, 1996). The rapid development of aerobic metabolic capacity observed during this period is consistent with the ecological characteristics of juvenile PBT. The juveniles exhibit rapid growth up to 1 year of age, with the cohorts born in the Pacific Ocean between April and July reaching a body size of 20–30 cm $L_{f}$ by August–September upon their migration to the Tosa Bay. The growth rate during this stage is particularly high compared to the later stage (Jusup et al., 2011), and the juveniles reach 40 cm $L_{f}$ by October. Concurrently, their diet shifts from zooplankton to more energy-rich prey, such as small fish (Shimose et al., 2013; Kitagawa and Fujioka, 2017), and their caudal fins undergo morphological changes, increasing aspect ratios to optimize for high-speed sustained swimming (Kitagawa and Fujioka, 2017). Given that high growth rates during the early stages play a crucial role in determining juvenile survival (Tanaka et al., 2006), the juvenile-specific development of aerobic capacity in the PBT during this stage likely plays a key functional role in the early ecology of PBT.

A recent study has provided ecological insights into the high scaling exponents of metabolic rate during the early life stages of fish (Norin, 2022). The relationship between metabolic rate and growth rate has long been recognized (Altringham and Block, 1997; Sogard, 1997), with species or individuals exhibiting higher metabolic rates often showing faster growth rates, provided they meet their dietary demands (Auer et al., 2015a; 2015b; 2015c). Since mortality rates are highest during the early life stages of fish, rapid growth is believed to enhance survival rate (Sogard, 1997; Norin, 2022). Therefore, the study hypothesized that the ontogenetic scaling of the metabolic rate in fish is a result of selective pressures associated with high mortality in early life stages (Norin, 2022). The eco-physiological features of PBT juveniles are considered to coincide with this concept.

### 4.5 Conclusions and perspectives

It has been known that tunas begin to exhibit higher body temperature than ambient water at fork lengths of 20–40 cm, but the development of heat production capacity and its contribution to the difference between body and water temperature at this stage has not been fully understood. By examining multiple traits related to heat-producing capacity in PBT juveniles, this study provides new insights into the ontogenetic patterns of heat production capacity and its physiological basis underlying the development of endothermic ability in PBT juveniles. Our findings demonstrate that the juvenile-specific high heat-production rate is critical during the early stages of endothermic development. The observed high heat-production rates during this stage contrast with the subsequent decline as the fish grow larger. This study elucidates the ontogenetic development of metabolic heat production in juvenile PBT and its role in the acquisition of endothermic capability.

However, although the results of the present study implied a developmental shift in the physiological state, further studies are needed to explore internal changes, particularly energetic dynamics through ontogeny, in natural environments. Pioneering studies have proposed the measurement of heart rate in bluefin tunas (Clark et al., 2008; Clark et al., 2010), and recent technological advances in data loggers enable the measurement of long-term heart rate in bluefin tuna (Rouyer et al., 2023). It is hoped that an increasing number of physiological traits measured using biologging techniques will clarify the developmental process from exothermic to endothermic attributes in tuna species.

## Data Availability

The original contributions presented in the study are included in the article/supplementary material, further inquiries can be directed to the corresponding author.
